# Established non-union of an operatively managed trans-scaphoid perilunate fracture dislocation progressing to spontaneous union

**DOI:** 10.1007/s10195-011-0143-1

**Published:** 2011-06-30

**Authors:** Hiren M. Divecha, Jon V. Clarke, Steven J. Barnes

**Affiliations:** Department of Orthopaedic Surgery, Inverclyde Royal Hospital, Larkfield Road, Greenock, PA16 0XN UK

**Keywords:** Trans-scaphoid perilunate fracture dislocation, Non-union, Spontaneous union

## Abstract

Perilunate dislocations and fracture dislocations represent uncommon and unusual injuries that are often missed at initial presentation and diagnosed late in up to 25% of cases. Prompt open reduction, carpal stabilisation and ligamentous repair is required to reduce the risk of complications. We report a case of an established scaphoid non-union in an operatively managed perilunate fracture dislocation that spontaneously united almost 2 years after the initial injury, just before a planned revision scaphoid fixation with bone grafting. This case highlights the importance of initial clinical assessment together with appropriate radiographs and follow-up of these injuries post-operatively, especially when complications such as non-union arise.

## Background

Perilunate fracture dislocations represent uncommon and unusual injuries that are often missed at initial presentation in up to 25% of cases [1]. A neglected injury of this type can result in progressive carpal instability with ensuing post-traumatic arthritis, leading to pain and dysfunction [1]. Prompt open reduction, carpal stabilisation and ligamentous repair is required to reduce the risk of complications.

We report a case of an established scaphoid non-union in an operatively managed perilunate fracture dislocation that spontaneously united almost 2 years after the initial injury, just before a planned revision scaphoid fixation with bone grafting. To our knowledge, and following an extensive literature search of the PubMed and MEDLINE databases, such a case has not been reported on before.

## Case report

**Fig. 1:**
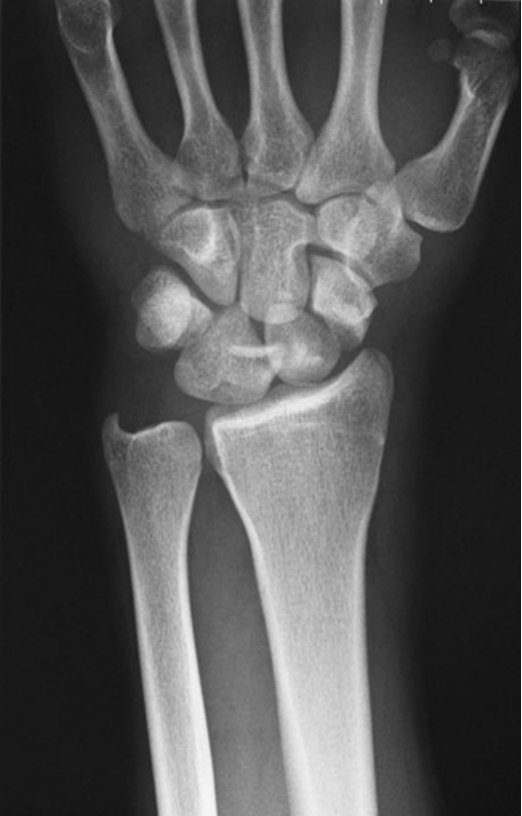
Wrist PA X-ray at presentation showing a displaced scaphoid fracture with disruption of Gilula’s arcs (1 and 2)

A 19-year-old, right-hand-dominant, smoker attended the emergency department with left wrist pain and swelling following a fall down a staircase. His wrist plain radiographs (Fig. 1) were interpreted as showing a radial styloid fracture, and he was managed in a below-elbow back slab and referred to the fracture clinic.

**Fig. 2:**
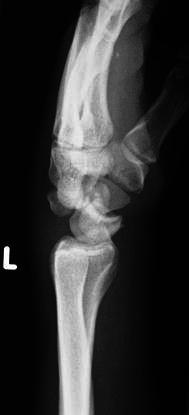
Wrist lateral X-ray showing volar perilunate dislocation

**Fig. 3:**
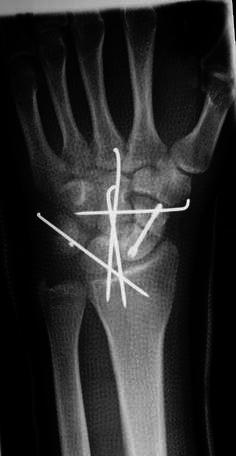
Wrist PA X-ray taken post-operatively

At review in the fracture clinic the next day, his radiographs were correctly interpreted with an additional true lateral wrist view (Fig. 2) as showing a trans-scaphoid perilunate fracture dislocation. He had symptoms of median nerve compression at the wrist. Computed tomography (CT) scan was not performed, and he underwent immediate surgical management. This involved a volar incision to decompress the carpal tunnel, reduce the lunate and repair the rent in the volar capsule. Through a dorsal approach, the scaphoid waist fracture was reduced and fixed with a Herbert screw. K-wire stabilisation of the carpal bones was performed, followed by a triquetro-lunate ligament repair using a Mitek suture anchor (Fig. 3). His median nerve symptoms resolved post-operatively, and he was discharged home in a below-elbow cast. The K-wires were removed at 4 weeks and he was placed into a wrist splint.

**Fig. 4:**
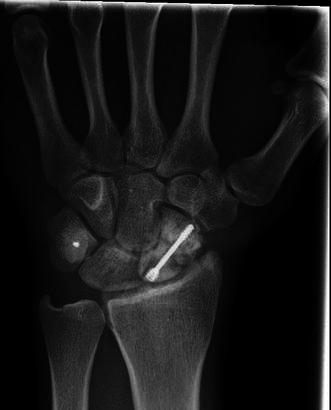
Wrist PA X-ray at 12 months suggesting non-union of scaphoid fracture

Unfortunately he failed to attend any further follow-up and was re-referred 12 months later with on-going pain at the base of the thumb. He had scaphoid tenderness on palpation in the ‘anatomical snuff box’ but also pain on radial deviation and a reduced range of movement (flexion 20°, extension 30°). Radiographically, he had features of a scaphoid non-union (Fig. 4). It was elected to proceed to revision of the scaphoid fixation with cortico-cancellous bone grafting for this non-union. CT scan was not performed as it was felt that the combination of scaphoid tenderness and radiological appearances supported the diagnosis of scaphoid non-union. Furthermore, his clinical symptoms warranted surgical intervention at this stage, nearly 13 months post injury.

**Fig. 5:**
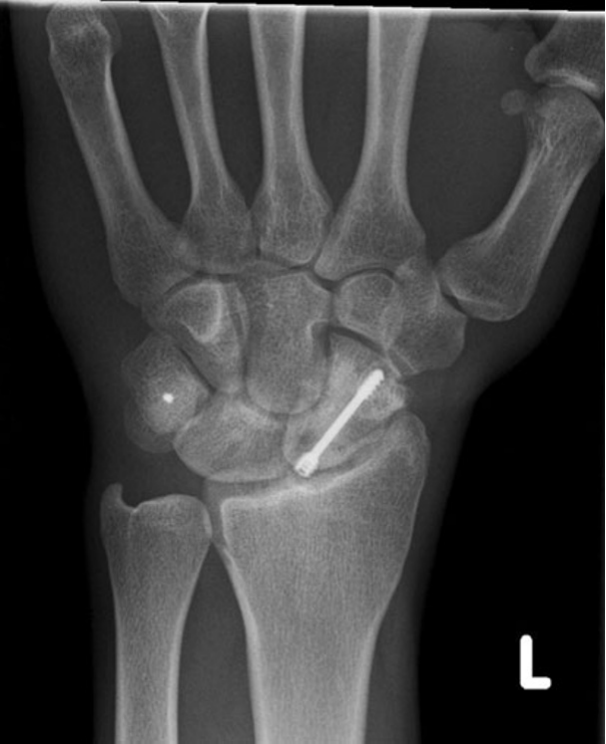
Wrist PA X-ray at 20 months suggesting scaphoid fracture union

On the day of his revision operation, a repeat X-ray (Fig. 5) was performed as he was no longer tender over the scaphoid. This suggested union of the scaphoid fracture (20 months post injury). The Herbert screw head appeared to have backed out slightly. He did however complain of pain on radial deviation of the wrist, attributed to radial styloid impingement on the scaphoid. Intra-operatively, the scaphoid fracture was united. The Herbert screw was loose and thus removed. A radial styloidectomy was performed to address the impingement, and at his last follow-up (6 weeks post-operative) his symptoms were settling and his wrist mobility was improved, though with some on-going stiffness. Unfortunately, he failed to attend any further follow-up.

## Discussion

Stability of the carpal bones is dependent on the perilunar ligaments and the scaphoid bone, which acts as a bony bridge between the proximal and distal carpal rows. Malgaigne was the first to describe the perilunate fracture dislocation, in 1855. These are uncommon injuries and represent 10% of all carpal injuries [2]. Lesser arc injuries are purely ligamentous and result in perilunar injury, with lunar dislocation being the last stage. Transosseous variants are classified as greater arc injuries and represent two-thirds of perilunar dislocations. They result in perilunar injury with fractures of the scaphoid, capitate, radial/ulnar styloid or triquetrum, or combinations thereof. The trans-scaphoid perilunate variant is reported to represent 61% of all perilunate fracture dislocations [1].

The general mechanism of injury results from hyperextension with ulnar deviation and intercarpal supination, as described by Mayfield et al. [3] in 1980. They suggested four stages of ligamentous disruption, progressing in a radial to ulnar direction, with volar lunate dislocation (into the space of Poirier) being the final stage.

Although prompt surgical intervention can potentially avoid progressive carpal instability, it has been reported that over 50% of operatively managed patients can develop post-traumatic arthritis [1]. Closed reduction alone is associated with poor outcome in 27% of cases due to difficulties in attaining and maintaining anatomic reduction [4]. Prompt open reduction, carpal stabilisation and ligamentous repair is the treatment of choice to prevent this.

Theoretically, the risk of scaphoid non-union would be higher in the setting of perilunate fracture dislocation due to increased soft tissue damage at time of injury and increased carpal instability thereafter. In a series of 15 operatively managed trans-scaphoid perilunate fracture dislocations, 13 were fixed with Herbert screws. Three developed non-union and required further bone grafting [5]. In another series, 25 trans-scaphoid perilunate fracture dislocations were operatively managed and no non-unions developed at a mean of 44.6 months [6]. More recently, Forli et al. [7] reported retrospectively on a series that included seven trans-scaphoid perilunate fracture dislocations managed operatively with a minimum 10 years follow-up. Three out of seven had excellent or good Mayo wrist score, and four out of seven had radiographical evidence of degenerative changes at follow-up. They only encountered one scaphoid non-union. The authors re-enforce that poor reduction in these injuries leads to worse degenerative changes, however this did not always correlate with wrist function.

There have been no reports of spontaneous unions of scaphoid fractures in established non-union following operative fixation in the setting of perilunate fracture dislocation. We are aware of three case reports of established isolated scaphoid non-unions that have spontaneously united, one in a 19-year-old requiring fixation [8] and the other two in paediatric cases. Clarke et al. [9] report on an 11-year-old and Manak et al. [10] report on a 14-year-old, both with established scaphoid non-unions that proceeded to union at 2 years post injury.

The important messages that the presented case highlights are as follows. Firstly, perilunate injuries are difficult to diagnose and emergency department doctors should be aware of these injuries. A history of high-energy injury with axial loading of an extended wrist should raise suspicion of a perilunar injury. Full clinical evaluation of the wrist and upper limb, including neurological examination, together with appropriate good-quality X-rays is essential.

Secondly, the importance of pre-operative clinical and radiological re-assessment cannot be stressed enough. This is important to prevent possible morbidity associated with unnecessary procedures. In this case, the patient’s clinical symptoms were significant enough to warrant surgical intervention. However, this needed to address the radial-sided impingement as the scaphoid had spontaneously united.

Finally, regular follow-up of these injuries is important for monitoring of rehabilitation and checking for possible complications.
